# Successful implementation of a risk assessment and mitigation program to control bovine digital dermatitis at the herd-level

**DOI:** 10.1038/s41598-025-12093-5

**Published:** 2025-08-20

**Authors:** Jim Weber, Marina Hillen, Torsten Seuberlich, Andreas Fürmann, Corinne Gurtner, Jens Becker, Claudia Syring, Maria Welham Ruiters, Maher Alsaaod, Lina Mazurek, Gertraud Schüpbach, Adrian Steiner

**Affiliations:** 1 Clinic for Ruminants, Department of Clinical Veterinary Medicine, Vetsuisse Faculty, University of Bern, Bern, 3012 Switzerland; 2 Graduate School for Cellular and Biomedical Sciences, University of Bern, Bern, 3012 Switzerland; 3 Division of Neurological Sciences, Vetsuisse Faculty, University of Bern, Bern, 3012 Switzerland; 4 Institute of Animal Pathology, Department of Infectious Diseases and Pathobiology, Vetsuisse Faculty, University of Bern, Bern, 3012 Switzerland; 5 Vetsuisse Faculty, Veterinary Public Health Institute, University of Bern, Bern, 3097 Switzerland; 6 Present Address: Section for Poultry and Rabbit Diseases, Institute for Food Safety and Hygiene, Vetsuisse Faculty, University of Zurich, Zurich, 8057 Switzerland

**Keywords:** Cattle, Disease control, Lameness, Surveillance, Treponemes, Skin diseases, Bacterial infection

## Abstract

This nonrandomized clinical intervention study was designed as a prospective, multicenter group comparison to evaluate the efficacy of a risk assessment and mitigation program to control bovine digital dermatitis (BDD). The program was implemented over a 1-year period on 9 intervention (INT) farms and 10 control (CTR) farms. Mainstays of the program derived from results of a previous BDD risk factor analysis. All farms were visited monthly to assess within-herd BDD prevalences to perform risk assessments and to treat BDD lesions with salicylic acid paste. Bulk milk samples were collected every 4 months. Diagnosis of BDD was based on visual inspection (clinical scoring) of the feet. Risk-associated management practices were identified on each farm, and management changes expected to prevent further introduction or spread of BDD within INT farms were suggested and agreed upon with farmers of the INT farms. Lesional biopsies were taken from a subset of cows of the INT group before and 2 months after treatment for histopathological and molecular biological examination to confirm histological and bacteriological cure in addition to clinical cure. The initial BDD prevalences for the INT and CTR farms averaged 39.8% (IQR 16.2) and 41.0% (IQR 12.4) for overall BDD lesions, 25.9% (IQR 10.8) and 26.2% (IQR 14.5) for active BDD lesions, and 22.1% (IQR 6.9) and 23.7% (IQR 22.3) for chronic BDD lesions, respectively. After 1 year of implementation, overall BDD prevalences were reduced to 14.1% (IQR 8.2) on INT farms but remained at 41.6% (IQR 10.8) on CTR farms. A significant decline in bulk milk anti-*Treponema* antibodies over the 1-year period was found in INT as compared to CTR farms. Considering the results of the histopathological examination, of 16S metagenomic sequencing and of the Fluorescence *in situ* hybridization as indicators for healing, 6/7 (85.7%) selected lesions were cured 2 months post completion of treatment. The results of this study show that the described BDD control measures can markedly reduce the within-herd prevalence of BDD. The proposed procedure might provide the basis for a nationwide BDD mitigation program that could be of importance also beyond national borders.

**Supplementary Information:**

The online version contains supplementary material available at 10.1038/s41598-025-12093-5.

## Introduction

Bovine digital dermatitis (BDD) is an infectious disease of the foot skin which in its active phase is typically characterized by ulcerative painful lesions on the rear feet between the bulbs. BDD has become a major problem in nearly all countries with intensive dairy production, due to its contagious nature and poor treatment response^1–3^. Prevalences have been reported to be as high as 91% at the herd-level and 41% at the cow-level^4–8^. Affected animals may show lameness with resulting reduced food intake, milk yield, and fertility as well as increased culling rates, making BDD an important ruminant welfare issue with substantial economic implications^6,9–12^. Diagnosis of BDD is usually based on clinical scoring by visual inspection of the feet using the Mortellaro (M)-scoring system by Döpfer et al. (1997)^13^, amended by Berry et al. (2012)^14^. Lesions with an ulcerative character (i.e. M1, M2, M4.1) are referred to as active stages, whereas M3 and M4 lesions are commonly grouped as chronic stages^15^. Healed skin after a pre-existing lesion is referred to as M5^14^. In addition to clinical scoring, assessment of anti-*Treponema* antibodies in bulk milk samples via indirect ELISA might be a useful tool for herd screening since the occurrence of M2 stages is consistently associated with seroconversion^16–18^.

Even though the precise pathogenesis of BDD remains unclear and it is considered a multifactorial disease, there is strong evidence for *Treponema* spp. to be the central etiological agents^19–21^. Current treatment strategies primarily aim at limiting bacterial infection by topical or systemic application of antibiotics. Lincomycin and oxy- as well as chlortetracycline were successfully used in the past in the treatment of BDD, but the public opinion towards prudent use of antibiotics increases^22^ (Commission Notice – Guidelines for the prudent use of antimicrobials in veterinary medicine), mainly due to the increasing prevalence of antibiotic-resistant bacteria in human and veterinary medicine contexts^23^.

Salicylic acid (SA) has keratolytic, hyperemic, anti-inflammatory, and antimicrobial effects, which might facilitate the elimination of bacteria in deeper layers of the epidermis and promote epidermal repair by intensified desquamation. Its clinical efficacy in the treatment of BDD is well investigated^24–27^. In Switzerland and Germany, a commercially available paste formulation that combines SA with methyl salicylate is approved for treating hyperkeratotic skin disorders in cattle, horses, and sheep with a 1-day withdrawal period for meat and milk in cattle^26^.

Ample research has been performed over the past years to understand the epidemiology of BDD and to identify specific control measures enabling the reduction of BDD incidences within farms over time. This helped to develop a 5-point plan for disease control that was published as conference presentation in 2017^28^. Pedersen (2019)^29^ described a control program on a dairy herd encompassing 3 mainstays that focused on immediate treatment of active lesions, prevention, and ongoing monitoring. However, multicenter controlled studies about the effect on BDD prevalences at herd-level after the implementation of specific control measures are missing. In addition to the required visual inspection after successful treatment, assessing the absence of *Treponema* spp. in deeper epidermal layers is crucial when evaluating the true effect of such a control program, since chronic lesions are associated with the presence of pathogens still being present in the skin^13^.

Our objective was to evaluate the efficacy of a risk assessment and mitigation program (RAMP) over the course of 1 year in Swiss dairy farms focusing on treatment of affected animals and considering aspects of internal and external biosecurity. The effectiveness of BDD control was assessed by clinical prevalence monitoring and by determining the course of bulk milk antibodies over time.

We hypothesized that (i) the herd-level reduction of overall BDD lesions (OL), active lesions (AL), and chronic lesions (CL) within 1 year after the first implementation of this mitigation program will be significantly higher (≥ 50%) in intervention (INT) farms as compared to farms where no interventions have been mandated by the research group [i.e. control (CTR) farms], and (ii) INT farms will show a decline in bulk milk anti-*Treponema* antibodies over the 1-year period compared to CTR farms. Additionally, we aimed to explore whether clinical healing (i.e. M5) is also accompanied by histological as well as bacteriological cure.

## Results

### Descriptive statistical analyses

No significant differences between INT and CTR farms were observed at the beginning of the study regarding herd characteristics, such as herd size and apparent BDD prevalence (Table 1). Holstein Friesian was the predominant breed (INT, CTR: 86.3%, 92.4%); remaining breeds encompassed Brown Swiss, Swiss Fleckvieh, Simmental, Montbéliarde, and Jersey. In total, 9 INT and 10 CTR farms were included in the final analysis. Three INT farms had to be excluded due to a lack of compliance when implementing the recommended mitigation measures. Furthermore, 2 CTR were disqualified since there was a marked change in their BDD management during the study period. Supplementary Table 1 displays the reasons for exclusion in more detail.

**Table 1:** Description of the study population.

Item	Group		
	Intervention	Control	*P*-value
No. animals included	507	484	
Average herd size			0.76
Median	51	39	
Range	21–96	25–93	
Fluctuation of herd size (%)			0.68
Mean	3.5	8.6	
SD	3.1	7.6	
Apparent BDD prevalence at t_0_ (%)			
Overall lesions^1^			0.82
Median	39.8	41.0	
IQR	16.2	12.4	
Active lesions^2^			0.73
Median	25.9	26.2	
IQR	10.8	14.5	
Chronic lesions^3^			0.62
Median	22.1	23.7	
IQR	6.9	22.3	
Time t_0_–t_1_ (weeks)			0.49
Mean	23.3	23.5	
SD	1.3	1.9	
Time t_1_–t_2_ (weeks)			0.89
Mean	25	23.7	
SD	1.7	1.4	
305-day milk yield at t_0_ (kg)			0.79
Mean	8,589	8,163	
SD	942	1,216	
Housing	Freestall	Freestall	

^1^An animal was deemed affected by overall lesions if there was any clinical sign of bovine digital dermatitis (BDD) lesion present in ≥ 1 foot.

^2^An animal was deemed affected by active lesions if it showed any clinical sign of at least 1 active lesion in ≥ 1 foot, regardless of the simultaneous occurrence of any chronic lesion.

^3^An animal was deemed affected by chronic lesions if it showed clinical signs of at least 1 chronic lesion in ≥ 1 foot, regardless of the simultaneous occurrence of active lesions.

### Regression analyses

#### Apparent prevalence courses over time

**Fig. 1:**
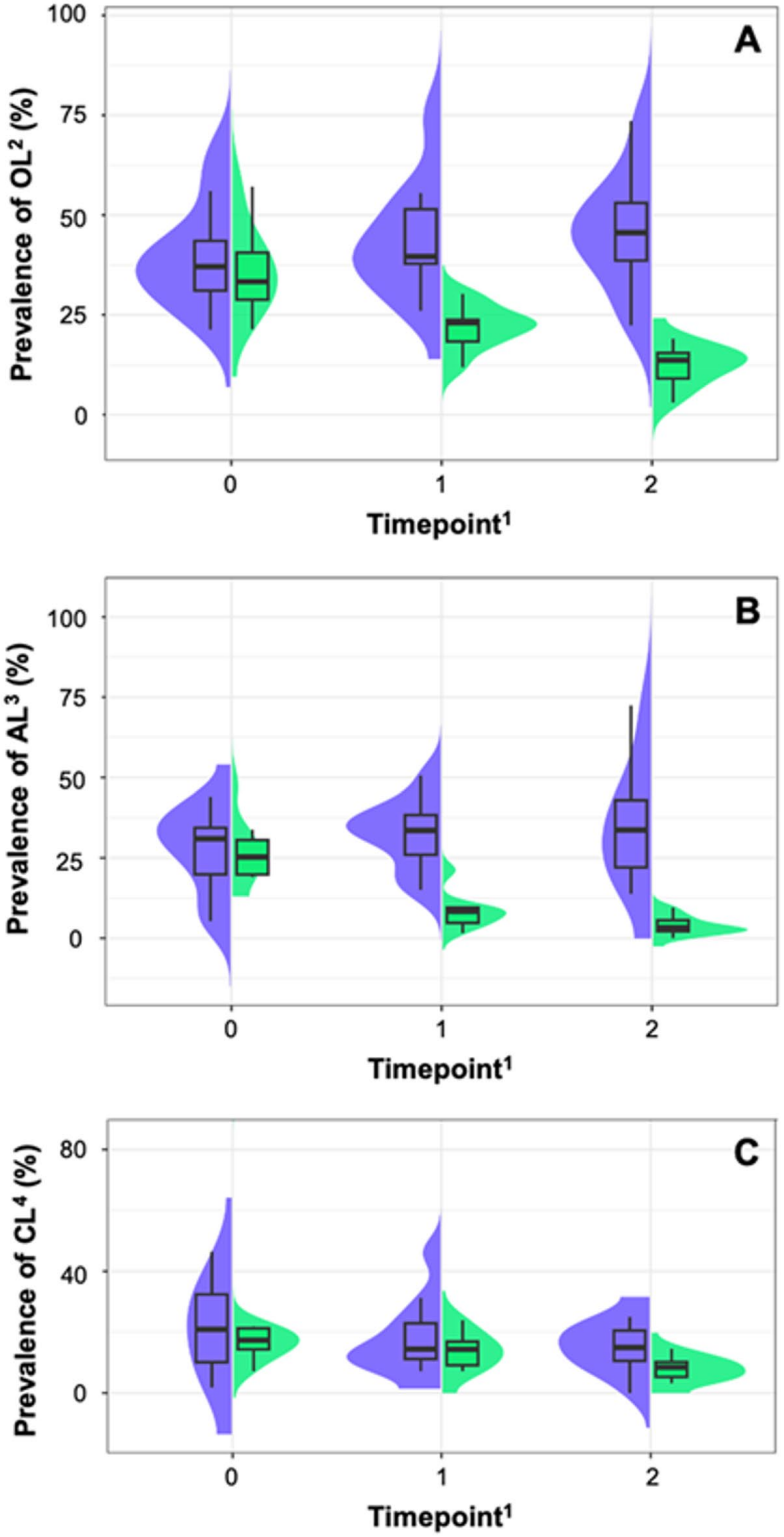
Apparent prevalence courses of bovine digital dermatitis (BDD) at cow-level over time during the 1-year study period: **(A)** overall BDD prevalence (OL); **(B)** prevalence of active lesions (AL); **(C)** prevalence of chronic lesions (CL). ^1^Timepoint t_0_ = 1 st claw trimming; t_1_ = 2nd claw trimming; t_2_ = 3rd claw trimming. ^2^An animal was deemed affected by OL if there was any clinical sign of BDD lesion present in ≥ 1 foot. ^3^An animal was deemed affected by AL if it showed any clinical sign of at least 1 AL in ≥ 1 foot, regardless of the simultaneous occurrence of any CL. ^4^An animal was deemed affected by CL if it showed clinical signs of at least 1 CL in ≥ 1 foot, regardless of the simultaneous occurrence of AL. blue = control farms; green = intervention farms; shades are showing the probability of observed data calculated by Kernel density estimation.

The courses of apparent within-herd BDD prevalences at the cow-level for OL, AL, and CL assessed in the trimming chute are displayed in Fig. 1. Table 2 shows the results of regression analysis. A significant difference between INT and CTR farms was observed over time. Implementation of control measures significantly reduced the occurrence of both OL and AL between t_0_–t_1_ and t_1_–t_2_ in INT as compared to CTR farms. Furthermore, there was a slight but non-significant reduction of CL within INT as compared to CTR farms between timepoints t_1_–t_2_. Monthly prevalence data collected between t_0_, t_1_ and t_2_ by the farmers or examiners of the research group in the milking parlor or when cows were restrained in head locks are shown in Supplementary Fig. 1. Prevalence courses between the claw trimmings were analyzed by descriptive statistics.

**Table 2:** Results of mixed effects regression analysis on the apparent prevalences of overall bovine digital dermatitis lesion prevalence, prevalence of active lesions, and prevalence of chronic lesions at the cow-level (*n*_INT_ = 507; *n*_CTR_ = 484).

Item	Odds ratio (95% CI)	*P*-value
Prevalence of overall lesions		
Intercept	0.50 (0.32–0.80)	0.004
Timepoint		
t_0_ (reference)	1	
t_0_–t_1_	1.36 (0.97–1.91)	0.079
t_1_–t_2_	1.50 (1.06–2.13)	0.023
Group		
CTR (reference)	1	
INT	0.81 (0.41–1.56)	0.522
Breed		
Holstein Friesian (reference)	1	
Others	0.78 (0.46–1.31)	0.347
Timepoint × group		
t_0_–t_1_		
CTR (reference)	1	
INT	0.26 (0.16–0.42)	< 0.001
t_1_–t_2_		
CTR (reference)	1	
INT	0.09 (0.05–0.16)	< 0.001
Prevalence of active lesions		
Intercept	0.20 (0.13–0.31)	< 0.001
Timepoint		
t_0_ (reference)	1	
t_0_–t_1_	1.66 (1.17–2.34)	0.004
t_1_–t_2_	2.15 (1.51–3.06)	< 0.001
Group		
CTR (reference)	1	
INT	1.21 (0.67–2.18)	0.525
Breed		
Holstein Friesian (reference)	1	
Others	0.83 (0.50–1.36)	0.456
Timepoint × group		
t_0_–t_1_		
CTR (reference)	1	
INT	0.09 (0.05–0.16)	< 0.001
t_1_–t_2_		
CTR (reference)	1	
INT	0.03 (0.02–0.06)	< 0.001
Prevalence of chronic lesions		
Intercept	0.17 (0.11–0.26)	< 0.001
Timepoint		
t_0_ (reference)	1	
t_0_–t_1_	0.80 (0.56–1.15)	0.231
t_1_–t_2_	0.43 (0.29–0.64)	< 0.001
Group		
CTR (reference)	1	
INT	1.09 (0.60–1.97)	0.775
Breed		
Holstein Friesian (reference)	1	
Others	0.81 (0.49–1.34)	0.418
Timepoint × group		
t_0_–t_1_		
CTR (reference)	1	
INT	0.70 (0.42–1.17)	0.174
t_1_–t_2_		
CTR (reference)	1	
INT	0.58 (0.32–1.03)	0.065

^1^An animal was deemed affected by overall lesions if there was any clinical sign of bovine digital dermatitis lesion present in ≥ 1 foot.

^2^An animal was deemed affected by active lesions if it showed any clinical sign of at least 1 active lesion in ≥ 1 foot, regardless of the simultaneous occurrence of any chronic lesion.

^3^An animal was deemed affected by chronic lesions if it showed clinical signs of at least 1 chronic lesion in ≥ 1 foot, regardless of the simultaneous occurrence of active lesions.

CTR = control group; INT = intervention group.

#### Anti-*Treponema* antibody titer courses in bulk milk over time

**Fig. 2:**
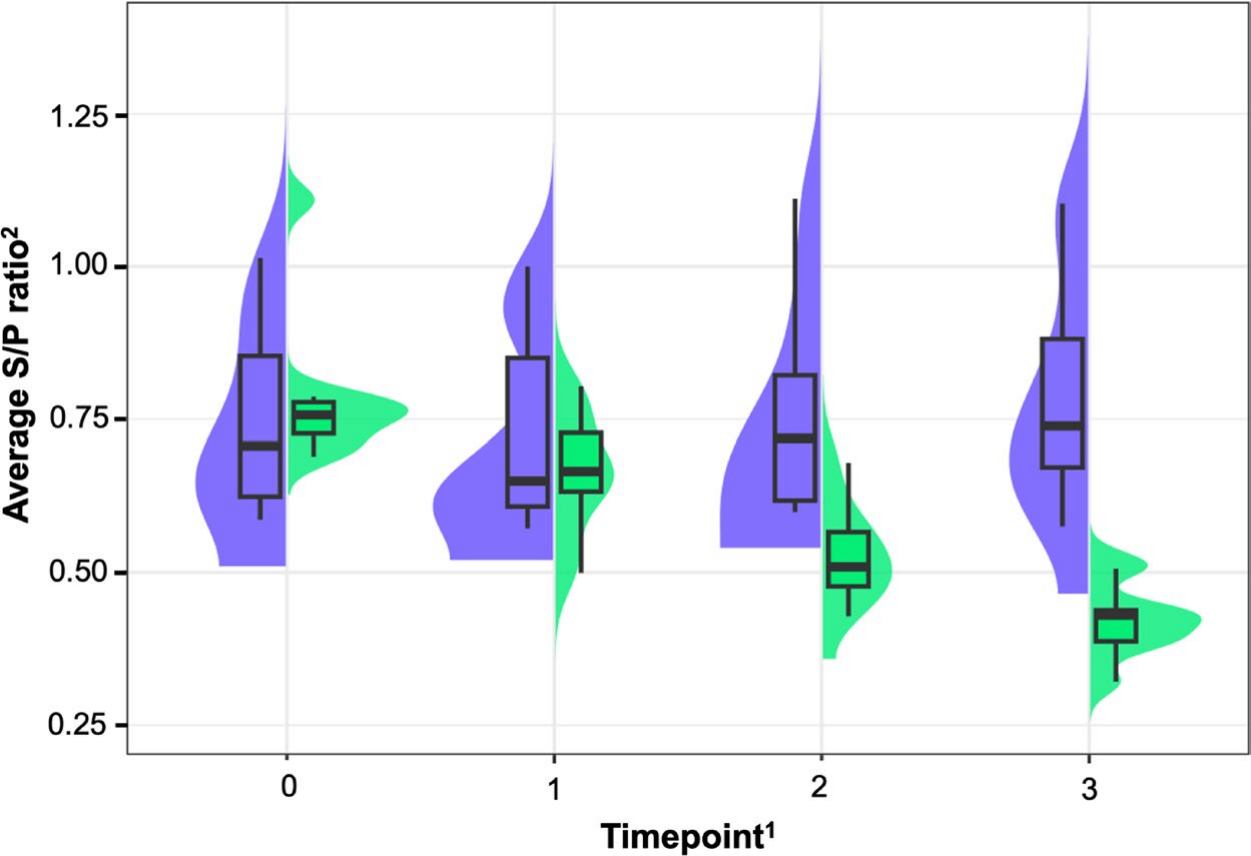
Course of bulk milk anti-*Treponema* antibodies over the 1-year study period in intervention (*n* = 9) and control farms (*n* = 10). ^1^Month after beginning of the study: t_0_ = month 0; t_1_ = month 4; t_2_ = month 8; t_3_ = month 12. ^2^Sample to positive ratio. blue = control farms; green = intervention farms; shades are showing the probability of observed data calculated by Kernel density estimation.

Bulk milk antibody courses are displayed in Fig. 2. Table 3 shows the results of linear regression analysis. We observed a significant difference between both groups over the 1-year observation period, when bulk milk samples were collected with an interval of 4 months. While there was a slight, but significantly (*P* = 0.044) higher decline in antibody titers expressed as sample to positive (S/P) ratio within INT compared to CTR farms between month 0 and month 4, the declines from month 4 to month 8 and from month 8 to month 12 were highly significantly (*P* ≤ 0.001) higher in INT as compared to CTR farms.

**Table 3:** Results of linear regression analysis on anti-*Treponema* antibody titer course of bulk milk samples, collected every 4 months over the 1-year study period in intervention (*n* = 9) and control farms (*n* = 10).

Item	Estimate (95% CI)	*P*-value
Intercept	0.75 (0.66–0.83)	< 0.001
Timepoint^1^		
t_0_ (reference)	1	
t_1_	−0.03 (−0.90–0.04)	0.450
t_2_	0.01 (−0.06–0.07)	0.837
t_3_	0.04 (−0.02–0.11)	0.212
Group		
CTR (reference)	1	
INT	0.04 (0.09–0.16)	0.551
Timepoint × group		
t_0_–t_1_		
CTR (reference)	1	
INT	−0.10 (−0.19–0.00)	0.044
t_1_–t_2_		
CTR (reference)	1	
INT	−0.26 (−0.36 – −0.17)	< 0.001
t_2_–t_3_		
CTR (reference)	1	
INT	−0.40 (−0.50 – −0.31)	< 0.001

^1^Month after beginning of the study: t_0_ = month 0; t_1_ = month 4; t_2_ = month 8; t_3_ = month 12.

CTR = control group; INT = intervention group.

### Biopsy samples – histopathological and bacteriological findings

**Fig. 3:**
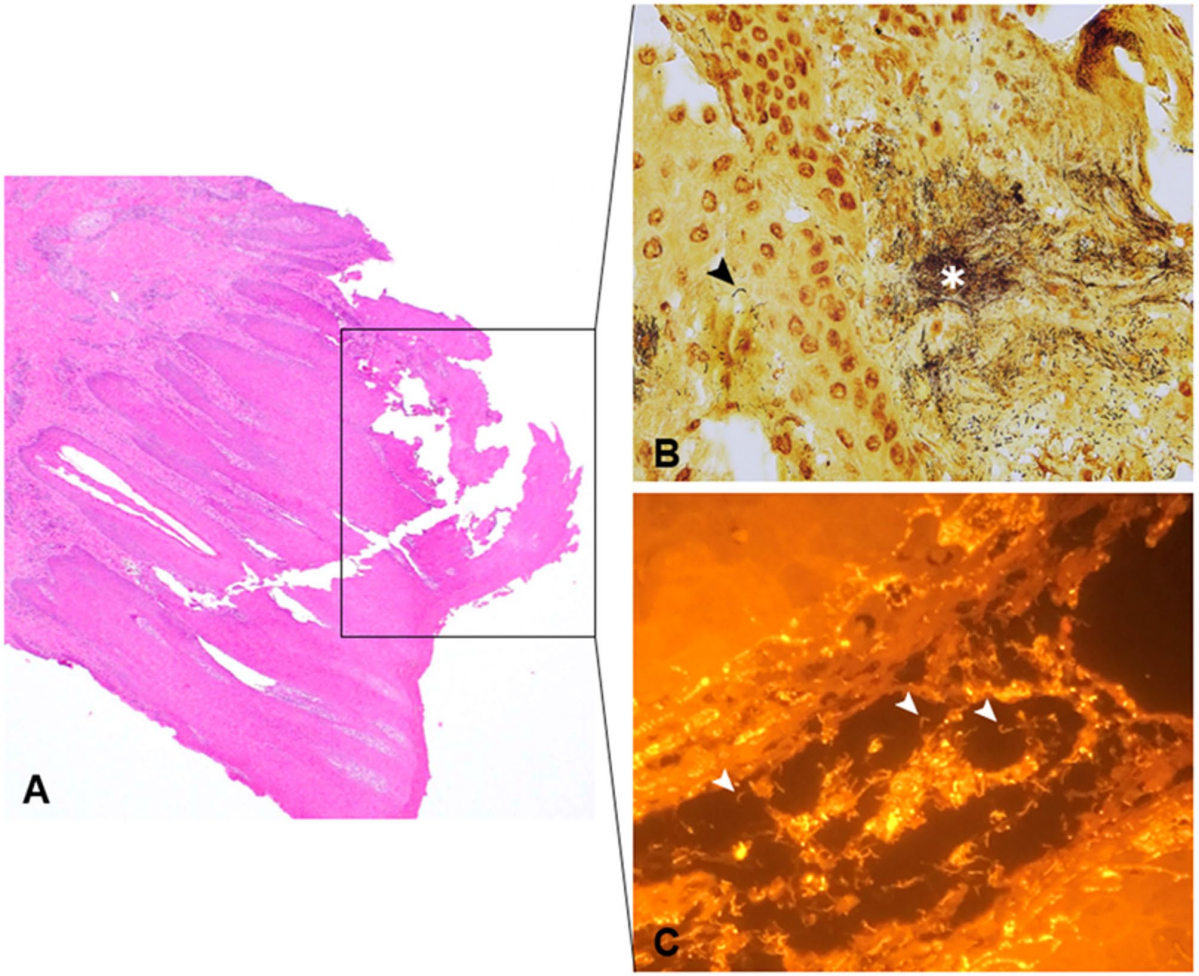
Histopathological appearance and findings of Fluorescence *in situ* hybridization (FISH) in a bovine digital dermatitis lesion biopsy graded as M2 according to Döpfer et al. 1997^13^: **(A)** keratolysis with ulceration of the epidermis (hematoxylin and eosin stain, magnification 20x); **(B)** high abundance (asterisk) of spirochetes within deeper layers of the epidermis (Warthin-Starry stain, magnification 40x); **(C)** deep infiltration of *Treponema* ssp. into the epidermis visualized by FISH (Cy3 labeled, magnification 40x). Arrowheads indicate spiral-shaped bacteria in (B) and (C).

Histopathological and 16S metagenomic findings are shown in Table 4. Figure 3 displays the findings in a M2 lesion biopsy before treatment, investigated by hematoxylin and eosin (HE) stain, Warthin-Starry (WS) stain, and Fluorescence *in situ* hybridization (FISH). A total of 33 lesional biopsies from 18 randomly selected cows (pre- and posttreatment) were available for histopathological examination, as 2 biopsies had to be discarded due to incomplete sampling and one cow revealed a recurrent M2 lesion 2 months after the end of the treatment (posttreatment; i.e. M5) where the second biopsy was not performed. The 16S metagenomic sequencing was conducted in 16/33 lesional biopsies, since this technique has not been available from the beginning of the study and because of poor DNA quality of 2 biopsies. All pretreatment biopsies were macroscopically characterized as being classic active ulcerative stages with corresponding, histopathological changes of moderate to severe perivascular, chronic, lymphoplasmacytic dermatitis. With the high-throughput sequencing (HTS) approach, *Treponema (T.) medium*, *T. pedis*, *T. phagedenis*, and *T. denticola* were detected.

**Fig. 4:**
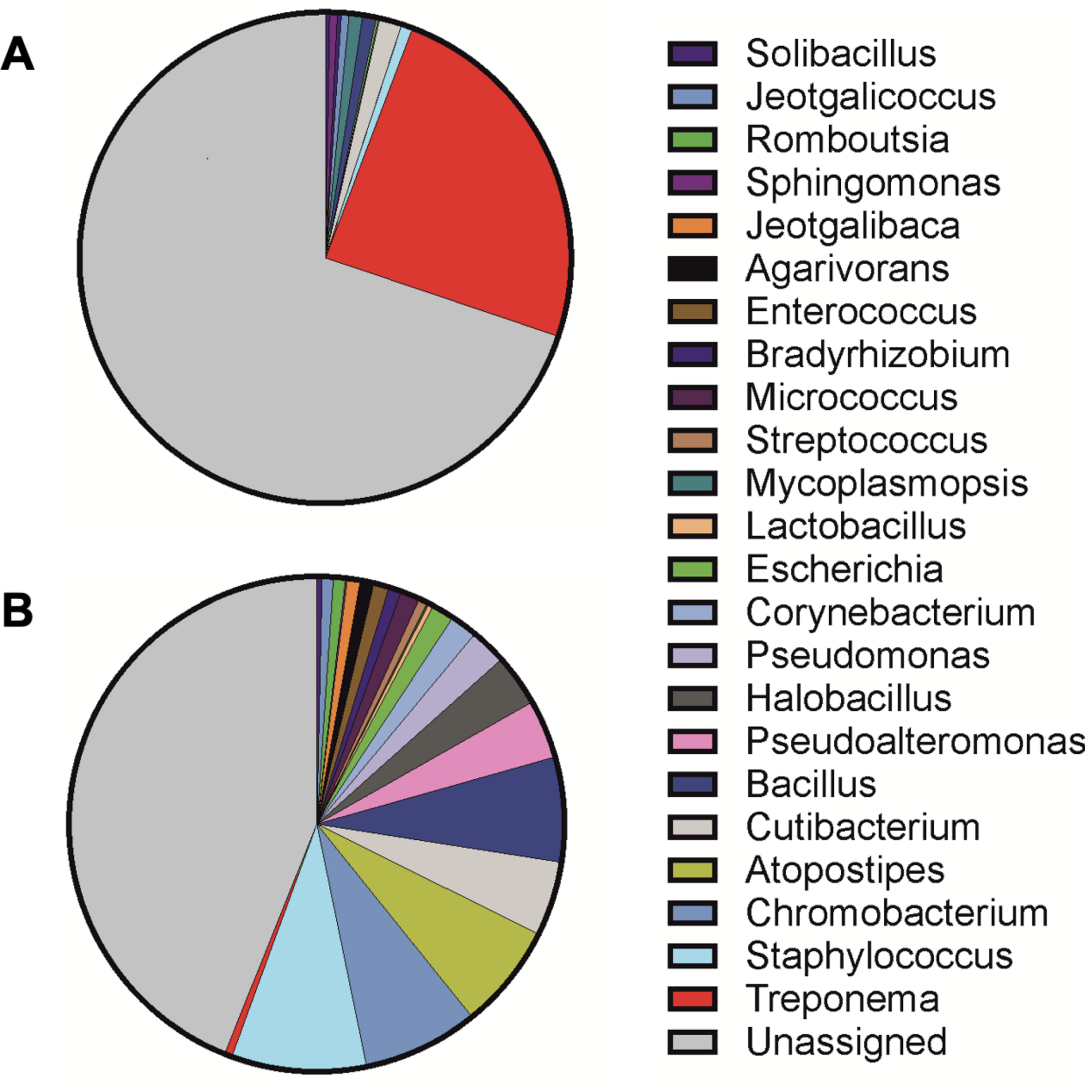
Relative abundance of bacterial genera determined by 16S rRNA gene metagenomic sequencing. *Treponema* was the most prevalent genus detected in pretreatment biopsy samples (**A**; total no. of reads = 20,723). After clinical cure, an increased diversity of bacterial genera with simultaneous reduction of *Treponema* spp. was observed (**B**; total no. of reads = 15,341). The number of total reads reflects the varying quantity of 16S rRNA in the samples.

When combining histopathological findings and results of FISH as prerequisites for healing, in 6/9 (66.7%) cows we did not observe histopathological alterations 2 months posttreatment. Considering results of the histopathological examination, the 16S metagenomics and FISH as prerequisites for healing, 6/7 (85.7%) cows were healed 2 months posttreatment. The mean relative abundance of bacterial genera assessed in biopsies collected before and after treatment is shown in Fig. 4. *Treponema* spp. was the most prevalent genus detected in pretreatment samples (24.4%). After clinical cure, the proportion of *Treponema* spp. decreased to 0.5% with a simultaneous increase of bacterial diversity. The diversity in the composition of bacterial genera tested within overall pretreatment samples was numerically lower compared to the diversity within overall posttreatment samples (i.e. alpha diversity; Shannon’s index pretreatment, posttreatment: 0.88, 2.15). Furthermore, we observed a moderate difference in the composition of bacterial genera between overall pretreatment and posttreatment samples (i.e. beta diversity; Bray–Curtis dissimilarity: 0.58)^30^.

**Table 4:** Results of histopathological as well as molecular biological investigations of biopsy samples collected pre- and posttreatment from a subset of cows of intervention farms to evaluate treatment success and BDD-associated pathogens.

		Histopathological findings						Bacteriological findings (16S metagenomics)		Results of FISH^1^ analysis	
	Timepoint^2^	Pre	Post	Pre	Post	Pre	Post	Pre	Post	Pre	Post
	Item	Dermatitis/keratolysis score (0–3)^3^		Ulceration score (0–1)^4^		Spirochetal load score (0–3)^5^		Detected ***Treponema*** species^6^ (no. of 16S rRNA gene reads per 10,000 total reads)		Treponemal load score (0–3)^7^	
**Cow #**	1	2/2	1/0	1	0	1	0	n.p.	n.p.	2	0
	2	3/1	1/0	1	0	2	0	n.p.	n.p.	3	0
	3	3/2	1/0	1	0	3	0	n.p.	n.p.	2	0
	4	2/1	n.u.	1	n.u.	2	n.u.	n.p.	n.p.	2	n.u.
	5	2/1	1/0	1	0	2	0	n.p.	n.p.	2	0
	6	2/0	1/0	0	0	1	0	n.p.	n.p.	3	0
	7	2/2	3/0	1	1	2	0	n.p.	n.p.	3	1
	8	2/0	0/0	0	1	1	0	n.p.	n.p.	2	0
	9	2/1	3/1	1	1	2	1	n.p.	n.p.	2	2
	10	2/1	n.a.	1	n.a.	1	n.a.	n.p.	n.p.	3	n.a.
	11	3/1	1/0	1	0	1	0	*T. medium* (150), *T. phagedenis* (830)	-	2	0
	12	n.u.	1/0	n.u.	0	n.u.	0	n.u.	*T. phagedenis* (4)	n.u.	0
	13	2/2	1/0	1	0	1	0	*T. phagedenis* (1,109)	*T. phagedenis* (12)	2	0
	14	3/2	3/0	1	1	2	0	*T. medium* (22), *T. phagedenis* (198)	*T. medium* (19), *T. phagedenis* (45), *T. denticola* (15)	2	1
	15	2/2	1/0	1	0	3	1	*T. medium* (19), *T. phagedenis* (110), *T. denticola* (95)	n.u.	2	0
	16	2/0	0/0	1	0	1	0	*T. medium* (7), *T. phagedenis* (202), *T. pedis* (976), *T. denticola* (19)	-	2	0
	17	2/1	1/0	1	0	1	0	*T. phagedenis* (741), *T. pedis* (452)	-	1	0
	18	3/1	2/0	1	0	3	0	*T. medium* (318), *T. phagedenis* (1,086), *T. denticola* (181)	-	3	0

^1^Fluorescence *in situ* hybridization; Cy3 labeled for *Treponema (T.)* spp.

^2^Pre = pretreatment; post = posttreatment at 2 months after clinical cure.

^3^Chronic dermatitis was scored as follows: 0 = no histological changes; 1 = mild, perivascular, chronic, lymphoplasmacytic dermatitis; 2 = moderate perivascular, chronic, lymphoplasmacytic dermatitis; 3 = severe, perivascular, chronic, lymphoplasmacytic dermatitis; keratolysis was scored as follows: 0 = none; 1 = focal; 2 = multifocal; 3 = diffuse^31,32^.

^4^Ulceration was scored as follows: 0 = none; 1 = present^31^.

^5^Amount of spirochetes detected by Warthin-Starry stain was scored as follows: 0 = no visible spirochetes; 1 = minimal amount; 2 = moderate amount; 3 = high amount^31^.

^6^Samples yielding < 10,000 reads were declared as not usable due to insufficient DNA quality.

^7^Hybridization signal for *Treponema* spp. detected by FISH was scored as follows: 0 = no hybridization; 1 = sparse hybridization; 2 = moderate hybridization; 3 = strong hybridization^32^.

n.a. = not applicable (i.e. no clinical cure); n.p. = analysis not performed since methodology was not available until the middle of the study; n.u. = not usable.

## Discussion

We were able to markedly reduce prevalences of OL and AL in intervention farms within 1 year by implementing farm-specific control measures targeting internal and external biosecurity and effective treatment of occurring acute lesions compared to a monitored control group. Lack of histopathological findings and bacteriological detection of *Treponema* spp. were observed in most treated animals. However, eradication of the disease at the herd-level was not achieved. The assessment of bulk milk antibody courses was shown to be a useful additional tool for monitoring the efficacy of this program. Since BDD within-herd prevalences of both study groups were quite equal at the beginning of the trial, the significant reduction in the occurrence of BDD over time can be attributed to our intervention measures. To our knowledge, this is the first multicenter controlled study investigating the efficacy of a RAMP for BDD in dairy cattle.

The implemented measures were based on a risk assessment strategy that was developed based on previously published literature, discussion within the research group and the opinion of external experts in the field^28,33,34^. The mitigation measures for the intervention farms were grouped into “mandatory” and “voluntary” based on feasibility, dictated by the farm management characteristics. Therefore, culling of recurrently infected animals and prevention of overcrowding were considered voluntary mitigation measures. The installation of a footbath was not implemented on any of the farms. Therefore, it was replaced by topical application of claw disinfectants, as an adequate alternative to footbathing^35^. The present study does not allow to evaluate the effect of individual measures, since all farms included in the final analyses had to comply with a list of several measures.

Combining the results of prevalence assessment by clinical scoring with serological analyses is a new approach when evaluating the efficacy of a RAMP in buiatrics. It might enhance the level of evidence regarding disease surveillance. Clinical scoring of animals restrained in the trimming chute is considered the gold standard of BDD diagnosis, because this is the most sensitive of the clinical methods^36^. Consequently, only prevalence data collected during the biannual claw trimmings were included in our final analyses. To monitor the occurrence of BDD cases in a regular monthly manner over the 1-year study period, we used a modified scoring method according to Cramer et al. (2018)^36^ to minimize the amount of labor. Pre-washed feet were examined using a flashlight by walking through the pen when animals were restrained in the headlocks. Assessment of the bodyweight distribution and thorough inspection of animals showing abnormal hind-limb position with approx. 50 to 100 cm between the observer and the cow allowed to identify most active lesions that are crucial in disease transmission^15^. Therefore, the pen walk described can be recommended for the monitoring of such a RAMP.

Previous studies showed the usefulness of an indirect ELISA using bulk milk samples as an additional tool to diagnose BDD at the herd-level^18^. In the current study, the ELISA was conducted as described by Holzhauer et al. (2023)^17^ to primarily assess the presence of IgG anti-*Treponema* antibodies, revealing a solid test performance with a sensitivity of ~ 80% and a specificity of 100%. Our results are in line with the observations of Gomez et al. (2014)^16^ who found significantly lower antibody titers in treated animals that had suffered from M2 lesions compared to levels of those cows that did not undergo any treatment after an average of 220 days. The analysis of antibody titers in bulk milk, therefore, seems to be a useful option for the continuous surveillance of disease progress and the monitoring of BDD control programs. However, so far industry standard categories for high, medium and low prevalences of AL based on bulk milk antibody titers have not been published to the authors’ knowledge.

All biopsy samples were evaluated by both hematoxylin and eosin (HE) and Warthin-Starry (WS) staining. Since WS staining is rather unspecific for the detection of treponemes within deeper layers of the epidermis, additional analytical methods for the detection thereof are necessary. As described by Alsaaod et al. (2022)^26^ the combination of different molecular methods enhances both sensitivity and specificity when assessing bacteriological lesion cure. In this study, the FISH was targeted at *Treponema* spp., which did not allow to identify the bacteria at species-level. Therefore, we supplemented this method by full-length 16S rRNA gene high-throughput sequencing (HTS) and bioinformatics analysis. It only was performed in biopsies of 8 cows since HTS was available not earlier than from the middle of this study. Although the number of samples does not allow to perform statistically robust analyses, it might provide insights into the nature of bacterial compositions before and after treatment.

Sequencing of the full-length 16S rRNA gene produces reads ~ 1,400 base pairs, compared to much shorter sequences when targeting the variable gene regions (V3 and V4) commonly used Sanger sequencing or short-read HTS. Analysis of full-length 16S sequences results in increased taxonomic resolution and accuracy^37^. In addition, HTS based 16S metagenomics allows a quantitative assessment of bacterial communities, including *Treponema* spp., in a given sample. For HTS, there is no defined threshold number of reads required to consider a sample positive. Therefore, we focused on the comparison of the read number pretreatment vs. posttreatment. BDD-related treponemes can also be found as part of the microbiome in biopsy samples of clinically healthy skin^38^ probably due to environmental contamination. To differentiate between infection and colonization, histopathology and molecular methods were combined to assess the epidermal invasion depth of the treponemes. A reliable sign of infection is the invasion of treponemes into deeper layers of the epidermis^39^. Differentiating between new and latent, re-activated BDD infections remains challenging. In the present study, we decided to sample M2-affected animals 2 months after clinical lesion cure which allows to detect recurrence within this period, since a potential recurrence seems important within the scope of BDD control. Many aspects of both the pathogenesis and immunology of BDD remain unclear until now, including the possibility of intracellular invasion by the pathogens rather than purely extracellular presence. A possible infection of phagocytes with resulting escape of the bacteria from cellular immunity could be one explanation for the high rates of recurrence. The depth of tissue biopsies may thus not always be sufficient to detect treponemes.

The use of SA-based topical treatment represents a change towards a non-antibiotic alternative that addresses both infectious and inflammatory aspects of BDD. This approach aligns with the increasing emphasis on reducing antibiotic use in livestock management^22^.

We were not able to significantly reduce CL, due to the necessary discontinuation of lesion treatment upon the occurrence of exacerbations. As chronic lesions might reactivate, omission of CL treatment may have markedly contributed to incomplete elimination of BDD at the herd-level.

### Methodological strengths and limitations

The application of stringent inclusion criteria followed by a previous sample size calculation ensured robust results. Clinical prevalence data collected during routine herd claw trimmings were used as primary outcomes for the final analyses. Routine herd claw trimmings were defined as at least 80% of the herd being examined and trimmed^31^. This allows for the highest accuracy in data collection. The implementation of a multicenter study design enhanced the external generalizability of the findings while increasing the variability of the dataset. However, it has to be emphasized that herd sizes in Switzerland are small compared to other countries. This might impair the uncritical transfer of our observations to large-scale farms. Because of a selective dropout of INT farms that were not willing to implement the given mitigation measures or of CTR farms that substantially changed their BDD management, the risk of attrition bias must be considered. Since the inclusion of eligible study herds and their assignment to the respective study group (i.e. CTR vs. INT) were based on farmer’s preference, the present trial required a nonrandomized study design. This represents a potential risk for the occurrence of selection bias. Interobserver variability was tried to be kept low, involving only 2 observers that had to achieve an acceptable agreement of 80% at the beginning of the study.

### Conclusions and outlook

The RAMP for control of BDD described in this paper was able to significantly reduce the prevalence of OL and AL. Success strongly depends on the farmer’s compliance with the suggested control measures. It must be highlighted that disease control is time-consuming and labor-intensive and must be balanced against the costs. Further studies are necessary to investigate economic aspects accompanying such a RAMP including potentially enhanced animal health and milk/meet production as well as decreased treatment costs and culling rates. Although the study conducted by Pedersen (2019)^29^ revealed similar results by implementing some related measures as we did, the present multicenter controlled study indicates for the first time that a marked reduction in BDD prevalences at the farm-level can be achieved within 1 year by implementation of the described control measures. Future research should focus on the genetics that were shown to have a significant impact on the cow’s risk for infection^40–42^. Therefore, exclusion from breeding of repeatedly affected or therapy-resistant cows seems to be crucial. Genomic selection towards amelioration of foot health is increasingly and successfully used in different countries such as Canada, United Kingdom, Denmark, Sweden, Norway, Finland, and Germany^3,43–45^. In Switzerland, genetic indices for BDD are recently available for Holstein Friesian, Braunvieh (Brown Swiss as well as Original Brown), Swiss Fleckvieh, and Simmental cattle (unpublished data). Searching for appropriate treatments of M4 lesions requires further research and is of substantial importance for effective disease control. Even if standard industry categories for anti-*Treponema* antibody titers in bulk milk are not yet established, screening of bulk milk serology can be a useful tool to complement clinical prevalence assessment. The suggested measures might potentially provide the basis for a nationwide BDD control program that could be of importance also beyond national borders.

## Materials and methods

Reporting of this study is conducted according to the “Transparent Reporting of Evaluations with Nonrandomized Designs (TREND)” guidelines^46^. All methods were approved by the ethical committee of the veterinary office of the canton of Bern, Switzerland (national no. 34587; cantonal no. BE26/2022) and performed in compliance with the “Animal Research: Reporting In Vivo Experiments (ARRIVE)” guidelines 2.0^47^.

### Study design and farm selection

We designed this nonrandomized clinical intervention study as a prospective multicenter group comparison (INT vs. CTR) to investigate the efficacy of a BDD control program. Sample size was calculated assuming a base-level BDD within-herd prevalence of 0.35 and a reduction of ≥ 50% in INT as effect size, with α = 0.05 and β = 0.8 in a 2-sided test, an intra class correlation coefficient for the herd effect of 0.1, and an expected average herd size of 25 animals. This resulted in a required minimum of 475 analyzable animals in each group.

**Fig. 5:**
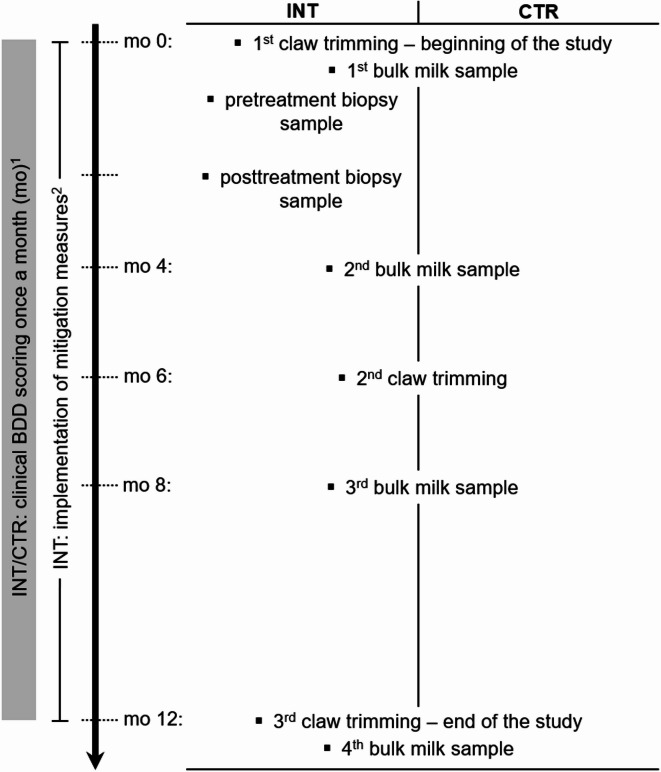
Study design: Timepoints of interventions, data and sample collection. ^1^Modified according to Cramer et al. (2018)^36^ with prior washing of cow’s feet. ^2^Regular and immediate treatment of BDD lesions until clinical cure as key component. BDD = bovine digital dermatitis; BM = bulk milk; CTR = control farms; INT = intervention farms.

Within a government-cofunded project to improve claw health in Switzerland (“Healthy claws – the foundation for the future”), data about the occurrence of disorders of the digits is continuously collected by professional claw trimmers via a digital recording software application, as described by Strauss et al. 2021^48^. Using these data, a list of farms revealing a BDD within-herd prevalence of ≥ 20% during the most previous claw trim, having a herd size of at least 20 lactating dairy cows, and performing a biannual preventive claw trimming of the entire herd was created. Eligible farms received a phone call with a short outline of the study and an invitation to participate. If they agreed, a pre-visit was conducted to explain the content of the project and asking for serving as a CTR or INT farm. Allocation to the groups was based on herd owners/managers’ preference and requirements set towards CTR compared to INT farms. They had to sign a contract agreeing to adhere to the recommended control measures described below (i.e. INT farms) or maintain their general management as usual (i.e. CTR farms). At the end of the study, the first author decided whether farmers or herd managers complied sufficiently with the intent of the program. Figure 5 provides an overview of the study design, conducted measures, and timepoints of data collection.

### Risk assessment and implementation of risk mitigation measures

On farms assigned to the INT group, risk factors for the establishment or transmission of BDD were thoroughly evaluated by filling in a checklist (Supplementary Table 2). Levels of risk were assessed using previously published scientific literature or by consideration of the opinion of experts within the study group and from the field. Depending on the outcome of the risk assessment, farm-specific best control measures were created. Herd managers that agreed to serve as INT farm had to adhere to the following measures of the RAMP addressing aspects to avoid introduction of BDD into the farm and to avoid the spread of the disease within the farm:

- Regular lameness monitoring once a week according to Sprecher et al. (1997)^49^ conducted by the farmers or the 2 examiners of the research group (AF, JW) during a pen walk or when cows exited the milking parlor;
- Regular screening for active BDD lesions every 14 days conducted by the farmers or examiners of the research group in the milking parlor or when cows were restrained in head locks;
- Recording and follow-up of BDD lesions;
- Cleaning and disinfection of claw trimming equipment after the treatment of a BDD-affected animal using powdered potassium monopersulfate (Virkon S, Arovet AG, Dietikon, Switzerland) that was dissolved in water at a concentration of 2% immediately before application^50^ (monitoring of the farmers’ compliance in regard to this measure was not feasible between the claw trimmings);
- Usage of single-use gloves when trimming animals with active lesions;
- Avoiding the sharing of claw trimming equipment between farms or cleaning and disinfection of claw trimming tools before entering on another farm;
- Screening of newly introduced animals into the farm for presence of BDD lesions in the trimming chute^51^;
- Control of re-introduced animals into the herd for presence of BDD lesions in the trimming chute;
- Immediate treatment of active as well as chronic M-stages^29,33^ whereby treatment of chronic lesions was stopped because of observed exacerbation (i.e. transition to an active character with focal ulceration and tenderness during palpation) in many cases;
- Footbathing or disinfection of feet using organic acidic solutions revealing low ecotoxicity with a concentration of 3–8% according to manufacturers’ instruction (all farms consistently used 1 product over the study period depending on farmers’ decision; i.e. EasyStride™, DeLaval AG, Sursee, Switzerland or Desintec® HoofCare Special D, Dr. E. Graeub AG, Bern, Switzerland);
- Minimization of manure exposure of the herd by adaption of a higher scraping frequency up to 8 times per day.

The aforementioned measures had to be newly introduced at entrance into the study on INT farms, or those measures already implemented had to be continued. Furthermore, it was recommended to voluntarily avoid over-crowding and to eliminate animals revealing recurrent BDD lesions despite initial clinical cure.

### Implementation of BDD control measures

The project started in August of 2022 and was completed in March of 2024. The beginning of the 1-year study period was the first routine claw trimming (t_0_) in all cases. Farms of the INT group were closely assisted by the research group with implementing the measures mentioned above via regular farm visits with an interval of at least one month. At each visit, all cows were scored for any BDD lesion by the research group when they were fixed in headlocks. Scoring was performed according to Cramer et al. (2018)^36^ with prior washing of animals’ feet.

Animals identified to suffer from any BDD lesion were treated without any delay at weekly intervals until clinical cure using 660 mg/g SA and 7.7 mg methyl salicylate paste (Novaderma ad us. vet., Streuli Tiergesundheit AG, Uznach, Switzerland), applied at a thickness of ~ 0.5 cm. The clinically healthy skin surrounding the lesion was protected from the keratolytic effect of SA by application of a thick coating of Milker’s fat cream (Eutra Tetina, Interlac, Puidoux, Switzerland)^26^. Feet were multi-layer bandaged including a water-repellent wound pad (Wundpolster, ITIN + HOCH GmbH, Liestal, Switzerland), cotton wool (Kistler Watte, Covetrus AG, Lyssach, Switzerland), cohesive tape (Cohesive bandages, Covetrus BV, Cuijk, The Netherlands), and water-resistant tape (tesa® Gewebeband, Covetrus AG, Lyssach, Switzerland). Visit frequency after the first and second routine claw trimming was once per week to support the farmer with bandage changes. Since exacerbations became apparent as described above in the majority of M4 lesions (50/56 distributed over all INT farms) after treatment, application of SA bandages to this lesion stage was stopped. Those lesions that aggravated were treated with povidone iodine ointment (Vetisept® ad us. vet., Dr. E. Graeub AG, Bern, Switzerland) under bandage to prevent complications like interdigital phlegmon. However, complete clinical cure could not be achieved in most of these cases. M3 lesions were treated throughout the study. Farms serving as CTR also received regular visits once a month, summing up to a total of 13 visits per farm. During these visits data were collected, and farmers were repeatedly inquired for potential changes in their BDD management. They received financial compensation after the end of the study.

### Data and sample collection, processing and analyses

#### Course of BDD prevalences

On both INT and CTR farms, BDD within-herd prevalences were determined at the cow-level during biannual, routine claw trimmings in the trimming chute by 2 examiners of the research group (AF, JW) and in between once a month when cows were restrained in headlocks or exited the milking parlor by the research group or farmers as follows: (i) animals were deemed affected by BDD in general if they revealed clinical signs of any BDD lesion stage in ≥ 1 foot; (ii) animals showing clinical signs of at least one AL (i.e. M1, M2, M4.1) in ≥ 1 foot were deemed affected for the assessment of active lesion prevalences, regardless of the potential occurrence of chronic lesions; (iii) cows with clinical signs of at least one chronic lesion (i.e. M3, M4) in ≥ 1 foot were considered as chronically affected, even if they suffered simultaneously from any active lesion.

Since 2 different examiners (AF, JW) collected prevalence data at the claw trimmings, a raw agreement of ≥ 80% had to be achieved taking interobserver reliability into consideration. For this purpose, all examiners assessed M-stages of one herd during the claw trimming in a blinded setting at the beginning of the study according to the scoring system described by Döpfer et al. (1997)^13^, modified by Berry et al. (2012)^14^. All data were transferred to Microsoft Excel (Office 2013, Microsoft Corp., Redmond, WA/USA).

#### Bulk milk sampling

Bulk milk samples were obtained every 4 months on both INT and CTR farms to investigate antibody courses over the 1-year observation period. All samples were collected using bronopol as preservative (10-ml milk sampling tubes, Suisselab AG Zollikofen, Zollikofen, Switzerland) and stored at − 20 °C until analysis. They were sent to the Royal GD Animal Health Laboratory (Deventer, The Netherlands) for processing and analysis.

#### ELISA

Bulk milk samples were analyzed using an indirect ELISA as described by Holzhauer et al. (2023)^17^. In brief, high-binding 96-well microplates (Greiner Bio-One, Alphen aan den Rijn, The Netherlands) were coated with a mixture of immunogenic proteins from *T. phagedenis*, *T. medium*, and 2 strains of *T. pedis* as whole-cell antigens. *Treponema*-specific antibodies were detected using horseradish peroxidase (HRP)-conjugated protein G (Abcam, Cambridge, United Kingdom) showing the highest affinity to IgG. Optical densities (OD) were assessed after unique testing using a microplate reader (Mikrotek, BioTek Instruments, Inc., Winooski, VT/USA) and S/P ratios were calculated using the following formula:

$$\:\varvec{S}/\varvec{P}=\frac{{\varvec{O}\varvec{D}}_{\varvec{s}\varvec{a}\varvec{m}\varvec{p}\varvec{l}\varvec{e}}-{\varvec{O}\varvec{D}}_{\varvec{n}\varvec{e}\varvec{g}\varvec{a}\varvec{t}\varvec{i}\varvec{v}\varvec{e}\:\varvec{c}\varvec{o}\varvec{n}\varvec{t}\varvec{r}\varvec{o}\varvec{l}}}{{\varvec{O}\varvec{D}}_{\varvec{p}\varvec{o}\varvec{s}\varvec{i}\varvec{t}\varvec{i}\varvec{v}\varvec{e}\:\varvec{c}\varvec{o}\varvec{n}\varvec{t}\varvec{r}\varvec{o}\varvec{l}}-{\varvec{O}\varvec{D}}_{\varvec{n}\varvec{e}\varvec{g}\varvec{a}\varvec{t}\varvec{i}\varvec{v}\varvec{e}\:\varvec{c}\varvec{o}\varvec{n}\varvec{t}\varvec{r}\varvec{o}\varvec{l}}}\:$$

#### Biopsy sampling

Biopsies of M2 lesions were taken before treatment and 2 months after clinical cure only in cases of M5 in a subsample of cows of INT farms (2 lesions per farm via stratified random sampling). BDD lesions were cleaned thoroughly with water and dried off with paper towels, followed by topical disinfection as described by Alsaaod et al. (2022)^26^. The foot was anesthetized using 2 different techniques depending on the localization of the lesion. If lesions were present within the interdigital cleft, a 2-point nerve block (20 mL of 2% lidocaine hydrochloride; Lidocain 2% Streuli ad us. vet., Streuli Tiergesundheit AG, Uznach, Switzerland) was applied^52^. In cases where lesions were located abaxially, an intravenous regional analgesia (Antalovsky block) was performed using the same medication as mentioned above^53^. Sampling and sample processing were done according to Alsaaod et al. (2022)^26^. In brief, 4-mm punch biopsies were taken and divided into 2 equal parts. One half was placed in 10% neutral buffered formalin for a maximum of 40 days until processing for histopathological examination. The other half was stored in an Eppendorf tube at − 20˚C until use for DNA extraction.

#### Histopathology and Fluorescence* in situ* hybridization (FISH)

Biopsy samples were routinely processed, paraffin-embedded and 4 μm slides were prepared. All sections were stained with hematoxylin and eosin (HE)^54^ and the Warthin-Starry stain (WS)^55^. Histopathological scoring of sections was conducted according to Read & Walker (1998)^31^ and adapted by Klitgaard et al. (2011)^32^ to evaluate the presence and severity of epidermal ulceration, keratolysis, acanthosis, epidermal as well as dermal changes, and bacterial colonization. Signs for treponeme-associated dermatitis include (i) focally circumscribed hyperplastic epidermis with or without parakeratotic proliferation, (ii) loss of stratum granulosum, (iii) present inflammatory response within the dermis. Keratolysis was assessed using the following score: 0 = none; 1 = focal; 2 = multifocal; 3 = diffuse. Chronic dermatitis was scored as follows: 0 = no visible changes; 1 = mild, perivascular, chronic, lymphoplasmacytic dermatitis; 2 = moderate, perivascular, chronic, lymphoplasmacytic dermatitis; 3 = severe, perivascular, chronic, lymphoplasmacytic dermatitis. The abundance of spirochetes evaluated by WS was classified as: 0 = nonvisible; 1 = minimal amount; 2 = moderate amount, 3 = high amount of spirochetes. For imaging, the Leica DMRB microscope was used (Leica Microsystems, Wetzlar, Germany).

FISH analysis was done as previously described by Alsaaod et al. (2022)^56^. The following oligonucleotide probe for *Treponema* spp. was used for FISH analysis: 5’-CAGAAACYCGCCTTCGCC-3’ (known as the S-S-TrepGenus-725 probe). In brief, hybridization was performed at 45 °C for 14 h with 100 µl of hybridization buffer. The final probe concentration was set to 5 ng/µl. After hybridization, the slides were washed with buffer, rinsed in water, dried, and mounted in Vectashield (Vector Laboratories Inc., Burlingame, CA/USA) for fluorescence microscopy. The probe was 5’ labeled with the isothiocyanate derivative Cyanine3 (Cy3; Eurofins MWG Operon, Ebersberg, Germany). To visualize the targeted fluorescent labeled proteins, an Axioimager M1 microscope was used. Images were obtained using an AxioCam MRm version 3 FireWire monochrome camera and AxioVision software, version 4.5 (Carl Zeiss, Oberkochen, Germany).

#### PacBio Full-length 16S rRNA Gene Amplicon Sequencing

DNA was extracted from skin biopsies using the Maxwell RSC DNA System (Promega Corporation, Madison, WI/USA). The starting DNA was assessed for quantity, quality and purity using a Qubit 4.0 fluorometer (Qubit dsDNA HS Assay kit; Q32851, Thermo Fisher Scientific, Waltham, MA/USA) and the Advanced Analytical FEMTO Pulse instrument (Genomic DNA 165 kb Kit; FP-1002-0275, Agilent, Santa Clara, CA/USA). Thereafter, the PCR amplification of bacterial full-length 16S rRNA genes (V1—V9 regions) was done according to the PN 101-599-700 procedure and checklist document from PacBio (London, United Kingdom) using primers.

5’-GCATC/barcode/AGRGTTYGATYMTGGCTCAG-3’ (known as the 27 F primer) and 5’-GCATC/barcode/RGYTACCTTGTTACGACTT-3’ (known as the 1492R primer). The barcodes are composed of 16 nt. The barcoded amplicons were pooled equi-volume, and a single-molecule real-time sequencing (SMRT) bell library was generated using the SMRTbell prep kit 3.0 procedure and checklist document PN 102-359-000 from PacBio.

SMRT sequencing was performed on the Sequel IIe instrument (PacBio) with the Sequel Sequencing kit 3.0, using a SMRT Cells 8 M with 2 h pre-extension followed by a 10 h movie time. Thereafter, the Circular Consensus Sequencing (CCS) generation was performed on the Sequel IIe to generate highly accurate single molecule reads (HiFi reads) and the barcode demultiplexing workflow was run in SMRT Link v11.1. All steps were performed at the Next Generation Sequencing Platform, University of Bern, Switzerland. HiFi reads were quality filtered with a cut-off set at Q20 and scaled to 10,000 reads per sample. The reads were then classified into bacterial taxa with the pb-16S-nf workflow (https://github.com/PacificBiosciences/HiFi-16S-workflow). Read counts per bacterial genus were extracted for samples before (pretreatment) and after (posttreatment). Only genera with total reads counts > 100 were further analyzed. Data were curated in Microsoft Excel (Office 2013, Microsoft Corp., Redmond, WA/USA) and Fig. 4 was generated by GraphPad Prism 10 (GraphPad, Boston, MA/USA). To estimate the diversity of bacterial genera within pre- and posttreatment samples (i.e. alpha diversity), Shannon’s index was used combining richness and eveness of each genus. Compositional differences in bacterial genera between pre- and posttreatment samples were measured by calculating the Bray–Curtis dissimilarity (i.e. beta diversity)^30^.

### Statistical analyses

Statistical analyses were performed using SPSS Statistics 25 (IBM Corp., Armonk, NY/USA) and R software (R: A language and environment for statistical computing, R Foundation for Statistical Computing, Vienna, Austria, https://www.r-project.org/). Categorical variables were described by frequency distributions, and continuous data were summarized by the mean ± standard deviation (SD) or median and range. Normal distribution was checked using histograms and the Shapiro-Wilk test.

Differences of characteristics between INT and CTR farms at the beginning of the study were tested using the Mann-Whitney U test, Chi-square test or Student’s t test, depending on how data were distributed and scaled.

To analyze the association between the occurrence of BDD at the cow-level as binary outcome at t_0_, t_1_, and t_2_ (i.e. biannual claw trimmings during the 1-year study period) and group (INT vs. CTR), mixed logistic regression models accounting for clustering at the herd-level and for repeated measures on the same animal were computed using the lme4 package in R. Three separate models were built for OL, AL, and CL, considering the following factors as fixed: timepoint (t_0_, t_1_, t_2_), group (INT vs. CTR), interaction timepoint × group, and breed. The individual cow was the experimental unit.

To evaluate the association between bulk milk antibody titers (i.e. S/P values) as metric outcome at t_0_, t_1_, t_2_, and t_3_ and group (INT vs. CTR), a mixed linear regression model was calculated, considering antibody titer of the farm as experimental unit, and accounting for repeated measures from the same farm. Timepoint, group and the interaction timepoint × group were set as explanatory variables. The predicted values were obtained from the models using fitted values with best linear unbiased estimates. Assumptions for this model were checked by normality test, Levene’s test and plots of residuals.

The goodness of model fit was assessed using the Akaike information criterion and by visual evaluation of residuals^57^. The level of significance was set at *P* ≤ 0.05.

## Electronic supplementary material

Below is the link to the electronic supplementary material.

Supplementary Material 1

Supplementary Material 2

Supplementary Material 3

## Data Availability

Data generated or analyzed during this study are included in this published article and its supplementary information files. High-throughput sequencing raw data are available from the National Center for Biotechnology Information (NCBI) short sequence archive (SRA) under the project no. PRJNA1272345 with accession no. SAMN48908227 to SAMN48908242.

## References

[1] Holzhauer, M., Hardenberg, C., Bartels, C. J. & Frankena, K. Herd- and cow-level prevalence of digital dermatitis in the Netherlands and associated risk factors. *J. Dairy. Sci.* **89**, 580–588 (2006). 16428627 10.3168/jds.S0022-0302(06)72121-X

[2] Capion, N., Thamsborg, S. M. & Enevoldsen, C. Prevalence of foot lesions in Danish Holstein cows. *Vet. Rec. ***163**, 80–85 (2008). 18641376 10.1136/vr.163.3.80

[3] Knappe-Poindecker, M. et al. Interdigital dermatitis, heel Horn erosion, and digital dermatitis in 14 Norwegian dairy herds. *J. Dairy. Sci.* **96**, 7617–7629 (2013). 24140335 10.3168/jds.2013-6717

[4] Rodriguez-Lainz, A., Melendez-Retamal, P., Hird, D. W. & Read, D. H. Papillomatous digital dermatitis in Chilean dairies and evaluation of a screening method. *Prev. Vet. Med. ***37**, 197–207 (1998). 9879592 10.1016/s0167-5877(98)00091-9

[5] Cruz, C., Driemeier, D., Cerva, C. & Corbellini, L. G. Bovine digital dermatitis in southern Brazil. *Vet. Rec. ***148**, 576–577 (2001). 11370885 10.1136/vr.148.18.576

[6] Relun, A., Lehebel, A., Chesnin, A., Guatteo, R. & Bareille, N. Association between digital dermatitis lesions and test-day milk yield of Holstein cows from 41 French dairy farms. *J. Dairy. Sci.* **96**, 2190–2200 (2013). 23415520 10.3168/jds.2012-5934

[7] Jury, A. et al. Prevalence of claw disorders in Swiss cattle farms. *Schweiz. Arch. Tierheilkd. ***164**, 779–790 (2021). 34758953 10.17236/sat00327

[8] Kofler, J. et al., Benchmarking Based on Regularly Recorded Claw Health Data of Austrian Dairy Cattle for Implementation in the Cattle Data Network (RDV)*. Animals (Basel) ***12**, 808 (2022). 10.3390/ani12070808 PMC8997101 35405797

[9] Losinger, W. C. Economic impacts of reduced milk production associated with papillomatous digital dermatitis in dairy cows in the USA. *J. Dairy. Res.* **73**, 244–256 (2006). 16569275 10.1017/S0022029906001798

[10] Nielsen, B. H., Thomsen, P. T. & Sorensen, J. T. A study of duration of digital dermatitis lesions after treatment in a Danish dairy herd. *Acta Vet. Scand. ***51**, 27 (2009). 19570191 10.1186/1751-0147-51-27 PMC2717074

[11] Evans, N. J., Murray, R. D. & Carter, S. D. Bovine digital dermatitis: Current concepts from laboratory to farm. *Vet. J. ***211**, 3–13 (2016). 27061657 10.1016/j.tvjl.2015.10.028

[12] Plummer, P. J. & Krull, A. Clinical perspectives of digital dermatitis in dairy and beef cattle. *Vet. Clin. North. Am. Food Anim. Pract.* **33**, 165–181 (2017). 28579041 10.1016/j.cvfa.2017.02.002

[13] Dopfer, D. et al. Histological and bacteriological evaluation of digital dermatitis in cattle, with special reference to spirochaetes and *Campylobacter faecalis*. *Vet. Rec. ***140**, 620–623 (1997). 9228692 10.1136/vr.140.24.620

[14] Berry, S. L., Read, D. H., Famula, T. R., Mongini, A. & Dopfer, D. Long-term observations on the dynamics of bovine digital dermatitis lesions on a California dairy after topical treatment with lincomycin HCl. *Vet. J. ***193**, 654–658 (2012). 22892182 10.1016/j.tvjl.2012.06.048

[15] Biemans, F., Bijma, P., Boots, N. M. & de Jong, M. C. M. Digital dermatitis in dairy cattle: the contribution of different disease classes to transmission. *Epidemics* **23**, 76–84 (2018). 29279186 10.1016/j.epidem.2017.12.007

[16] Gomez, A. et al. Immune response against Treponema spp. And ELISA detection of digital dermatitis. *J. Dairy. Sci.* **97**, 4864–4875 (2014). 24931522 10.3168/jds.2013-7616

[17] Holzhauer, M., Mars, J., Holstege, M. & van der Heijden, H. An in-house ELISA for *Treponema* antibodies in bulk milk as part of a monitoring tool for claw health in dairy herds. *Vet. Sci. ***10**, 571 (2023). 37756093 10.3390/vetsci10090571 PMC10537149

[18] Afonso, J. S. et al. Diagnosis of bovine digital dermatitis: Exploring the usefulness of indirect ELISA. *Front. Vet. Sci. ***8**, 728691 (2021). 34790712 10.3389/fvets.2021.728691 PMC8591176

[19] Evans, N. J. et al. Three unique groups of spirochetes isolated from digital dermatitis lesions in UK cattle. *Vet. Microbiol. ***130**, 141–150 (2008). 18243592 10.1016/j.vetmic.2007.12.019

[20] Wilson-Welder, J. H., Alt, D. P. & Nally, J. E. Digital dermatitis in cattle: Current bacterial and immunological findings. *Animals ***5**, 1114–1135 (2015). 26569318 10.3390/ani5040400 PMC4693204

[21] Kuhnert, P. et al. *Treponema phagedenis* (ex Noguchi 1912) Brumpt 1922 sp. nov., nom. rev., isolated from bovine digital dermatitis. *Int. J. Syst. Evol. Microbiol. ***70**, 2115–2123 (2020). 31999237 10.1099/ijsem.0.004027

[22] Commission Notice. – Guidelines for the prudent use of antimicrobials in veterinary medicine. (2015). https://health.ec.europa.eu/document/download/190841e8-5975-4390-a304-908c259592ab_en?filename=2015_prudent_use_guidelines_en.pdf

[23] Ariza, J. M., Relun, A., Bareille, N., Oberle, K. & Guatteo, R. Effectiveness of collective treatments in the prevention and treatment of bovine digital dermatitis lesions: A systematic review. *J. Dairy. Sci.* **100**, 7401–7418 (2017). 28668527 10.3168/jds.2016-11875

[24] Schultz, N. & Capion, N. Efficacy of salicylic acid in the treatment of digital dermatitis in dairy cattle. *Vet. J. ***198**, 518–523 (2013). 24268474 10.1016/j.tvjl.2013.09.002

[25] Capion, N., Larsson, E. K. & Nielsen, O. L. A clinical and histopathological comparison of the effectiveness of Salicylic acid to a compound of inorganic acids for the treatment of digital dermatitis in cattle. *J. Dairy. Sci.* **101**, 1325–1333 (2018). 29224873 10.3168/jds.2017-13622

[26] Alsaaod, M. et al. Proof of an optimized salicylic acid paste-based treatment concept of ulcerative M2-stage digital dermatitis lesions in 21 dairy cows. *PLoS One ***17**, e0269521 (2022). 35679311 10.1371/journal.pone.0269521 PMC9182225

[27] Weber, J., Richter, S. & Freick, M. Comparison of the therapeutic efficacy of salicylic acid paste with a polyurethane wound dressing for the treatment of digital dermatitis lesions in dairy cows. *Res. Vet. Sci. ***125**, 7–13 (2019). 31108427 10.1016/j.rvsc.2019.05.008

[28] Geldhof, J. et al. 5 point plan for control of Digital Dermatitis. *19th International Symposium and 11th Conference Lameness in Ruminants*, Munich, Germany, 152–153 (2017).

[29] Pedersen, S. Digital dermatitis control in the dairy herd: incorporating the ‘blitz’ treatment approach. *Livestock* **24**, 130–135 (2019).

[30] Kers, J. G. & Saccenti, E. The power of microbiome studies: Some considerations on which alpha and beta metrics to use and how to report results. *Front. Microbiol. ***12**, 796025 (2022). 35310396 10.3389/fmicb.2021.796025 PMC8928147

[31] Read, D. H. & Walker, R. L. Papillomatous digital dermatitis (footwarts) in California dairy cattle: Clinical and gross pathologic findings. *J. Vet. Diagn. Invest. ***10**, 67–76 (1998). 9526863 10.1177/104063879801000112

[32] Klitgaard, K., Boye, M., Capion, N. & Jensen, T. K. Evidence of multiple *Treponema* phylotypes involved in bovine digital dermatitis as shown by 16S rRNA gene analysis and fluorescence in situ hybridization. *J. Clin. Microbiol. ***46**, 3012–3020 (2008). 18562583 10.1128/JCM.00670-08 PMC2546760

[33] Weber, J. et al. Farm-level risk factors for digital dermatitis in dairy cows in mountainous regions. *J. Dairy. Sci.* **106**, 1341–1350 (2023). 36526455 10.3168/jds.2022-22243

[34] Ahlén, L., Holmoy, I. H., Nodtvedt, A., Sogstad, Å. M. & Fjeldaas, T. A case-control study regarding factors associated with digital dermatitis in Norwegian dairy herds. *Acta Vet. Scand* **64**, 19 (2022). 10.1186/s13028-022-00635-0 PMC9375421 35964105

[35] Fjeldaas, T., Knappe-Poindecker, M., Boe, K. E. & Larssen, R. B. Water footbath, automatic flushing, and disinfection to improve the health of bovine feet. *J. Dairy Sci. ***97**, 2835–2846 (2014). 24612817 10.3168/jds.2013-7531

[36] Cramer, G., Winders, T., Solano, L. & Kleinschmit, D. H. Evaluation of agreement among digital dermatitis scoring methods in the milking parlor, pen, and hoof trimming chute. *J. Dairy. Sci.* **101**, 2406–2414 (2018). 29290450 10.3168/jds.2017-13755

[37] Buetas, E. et al. Full-length 16S rRNA gene sequencing by PacBio improves taxonomic resolution in human Microbiome samples. *BMC Genom.* **25**, 310 (2024). 10.1186/s12864-024-10213-5 PMC10964587 38528457

[38] Moreira, T. F. et al. Pathology and bacteria related to digital dermatitis in dairy cattle in all year round grazing system in Brazil.* Plos One* **13**, e0193870 (2018). 10.1371/journal.pone.0193870 PMC5841792 29513739

[39] Nielsen, M. W. et al. Potential bacterial core species associated with digital dermatitis in cattle herds identified by molecular profiling of interdigital skin samples. *Vet. Microbiol. ***186**, 139–149 (2016). 27016768 10.1016/j.vetmic.2016.03.003

[40] Lai, E., Danner, A. L., Famula, T. R. & Oberbauer, A. M. Genome-Wide association studies reveal susceptibility loci for digital dermatitis in Holstein cattle. *Anim. (Basel) ***10**, 2009 (2020). 10.3390/ani10112009 PMC7693332 33142934

[41] Oelschlaegel, D. et al. Functional variants associated with CMPK2 and in ASB16 influence bovine digital dermatitis. *Front. Genet. ***13**, 859595 (2022). 35832195 10.3389/fgene.2022.859595 PMC9271848

[42] Tarsani, E. et al. Genome-wide association studies of dairy cattle resistance to digital dermatitis recorded at four distinct lactation stages. *Sci. Rep. ***15**, 8922 (2025). 40087373 10.1038/s41598-025-92162-x PMC11909109

[43] Anagnostopoulos, A. et al. Association between a genetic index for digital dermatitis resistance and the presence of digital dermatitis, heel Horn erosion, and interdigital hyperplasia in Holstein cows. *J. Dairy. Sci.* **107**, 4915–4925 (2024). 38331180 10.3168/jds.2023-24136 PMC11245669

[44] Heringstad, B. et al. Invited review: genetics and claw health: opportunities to enhance claw health by genetic selection. *J. Dairy. Sci.* **101**, 4801–4821 (2018). 29525301 10.3168/jds.2017-13531

[45] Malchiodi, F. et al. Symposium review: Multiple-trait single-step genomic evaluation for hoof health. *J. Dairy. Sci.* **103**, 5346–5353 (2020). 32331881 10.3168/jds.2019-17755

[46] Haynes, A. B., Haukoos, J. S. & Dimick, J. B. Trend reporting guidelines for nonrandomized/quasi-experimental study designs. *JAMA Surg. ***156**, 879–880 (2021). 33825826 10.1001/jamasurg.2021.0552

[47] du Percie, N. et al. Reporting animal research: explanation and elaboration for the ARRIVE guidelines 2.0. *PLoS Biol.* **18**, e3000411 (2020). 32663221 10.1371/journal.pbio.3000411 PMC7360025

[48] Strauss, G. et al. Evaluation of a novel training course for hoof trimmers to participate in a Swiss national cattle claw health monitoring programme. *Schweiz. Arch. Tierheilkd. ***163**, 189–201 (2021). 33650520 10.17236/sat00292

[49] Sprecher, D. J., Hostetler, D. E. & Kaneene, J. B. A lameness scoring system that uses posture and gait to predict dairy cattle reproductive performance. *Theriogenology* **47**, 1179–1187 (1997). 16728067 10.1016/s0093-691x(97)00098-8

[50] Gillespie, A. V., Carter, S. D., Blowey, R. W., Staton, G. J. & Evans, N. J. Removal of bovine digital dermatitis-associated treponemes from hoof knives after foot-trimming: A disinfection field study. *BMC Vet. Res. ***16**, 330 (2020). 32917195 10.1186/s12917-020-02552-8 PMC7488572

[51] Wells, S. J., Garber, L. P. & Wagner, B. A. Papillomatous digital dermatitis and associated risk factors in US dairy herds. *Prev. Vet. Med. ***38**, 11–24 (1999). 10022049 10.1016/s0167-5877(98)00132-9

[52] Nuss, K., Schwarz, A. & Ringer, S. [Local anaesthesia in ruminants]. *Tierarztl. Prax Ausg G Grosstiere Nutztiere* **45**, 159–173 (2017). 28508918 10.15653/TPG-161061

[53] Antalovsky, A. [Technique of intravenous local anesthesia in the distal limb in cattle]. *Vet. Med.* **7**, 413–420 (1965).

[54] Fischer, A. H., Jacobson, K. A., Rose, J. & Zeller, R. Hematoxylin and eosin staining of tissue and cell sections. *CSH Protoc* pdb.prot4986 (2008). 10.1101/pdb.prot4986 21356829

[55] Warthin, A. S. & Chronister, A. C. A more rapid and improved method of demonstrating spirochetes in tissues (Warthin and starry’s cover-glass method). *Am. J. Syph*. **4**, 97–103 (1920).

[56] Alsaaod, M. et al. Non-healing claw horn lesions in dairy cows: Clinical, histopathological and molecular biological characterization of four cases. *Front. Vet. Sci. ***9**, 1041215 (2022). 36337205 10.3389/fvets.2022.1041215 PMC9627347

[57] Akaike, H. Fitting autoregressive models for prediction. *Ann. Inst. Stat. Math. ***21**, 243–247 (1969).

