# Supplementary material for: Successful implementation of a risk assessment and mitigation program to control bovine digital dermatitis at the herd-level
Source: Sci Rep. 2025 Aug 20;15:30577. doi: 10.1038/s41598-025-12093-5 (PMC12368109; doi:10.1038/s41598-025-12093-5)
Supplement: Supplementary file 1 — Supplementary Material 1 [file 41598_2025_12093_MOESM1_ESM.pdf]

Supplementary Figure 1. Apparent prevalence courses of bovine digital dermatitis (BDD) at cow-level collected every month (t_0–t_{12}) during claw trimmings, fixation in headlocks or within the milking parlor during the 1-year study period: **A)** overall BDD prevalence (OL); **B)** prevalence of active lesions (AL); **C)** prevalence of chronic lesions (CL).

[1]Timepoint t_0 = 1st claw trimming; t_6 = 2nd claw trimming; t_{12} = 3rd claw trimming

[2]An animal was deemed affected by OL if there was any clinical sign of BDD lesion present in ≥ 1 foot.

[3]An animal was deemed affected by AL if it showed any clinical sign of at least 1 AL in ≥ 1 foot, regardless of the simultaneous occurrence of any CL.

[4]An animal was deemed affected by CL if it showed clinical signs of at least 1 CL in ≥ 1 foot, regardless of the simultaneous occurrence of AL.

blue = control farms; green = intervention farms; gray shades are showing data that were collected in the trimming chute

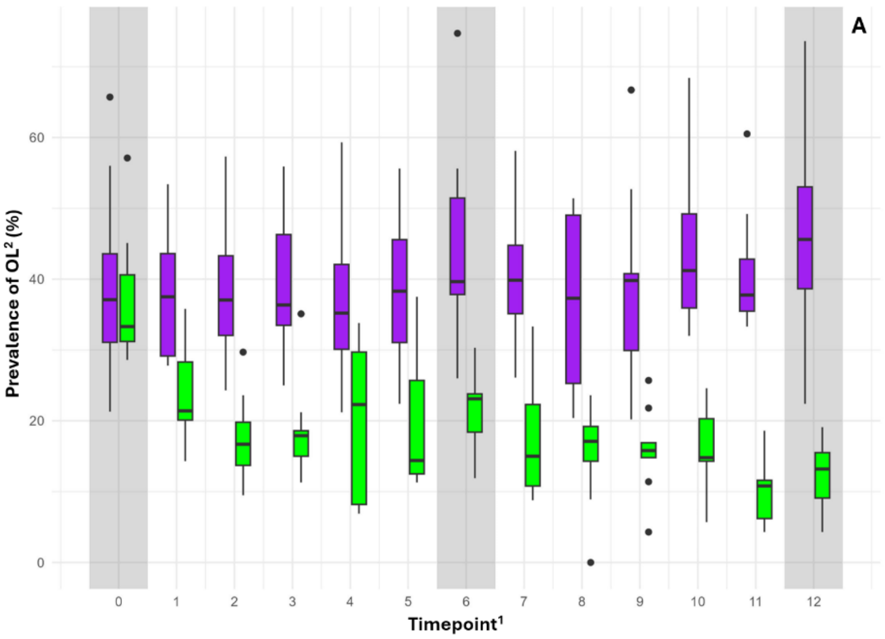
A
Prevalence of OL² (%)
0
20
40
60
0 1 2 3 4 5 6 7 8 9 10 11 12
Timepoint¹

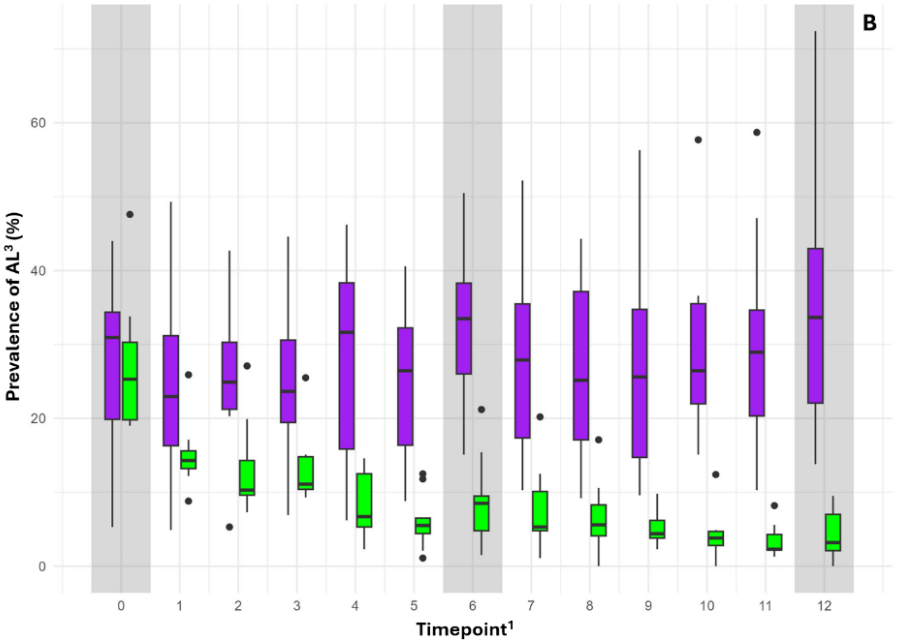
B
Prevalence of AL³ (%)
0
20
40
60
0 1 2 3 4 5 6 7 8 9 10 11 12
Timepoint¹

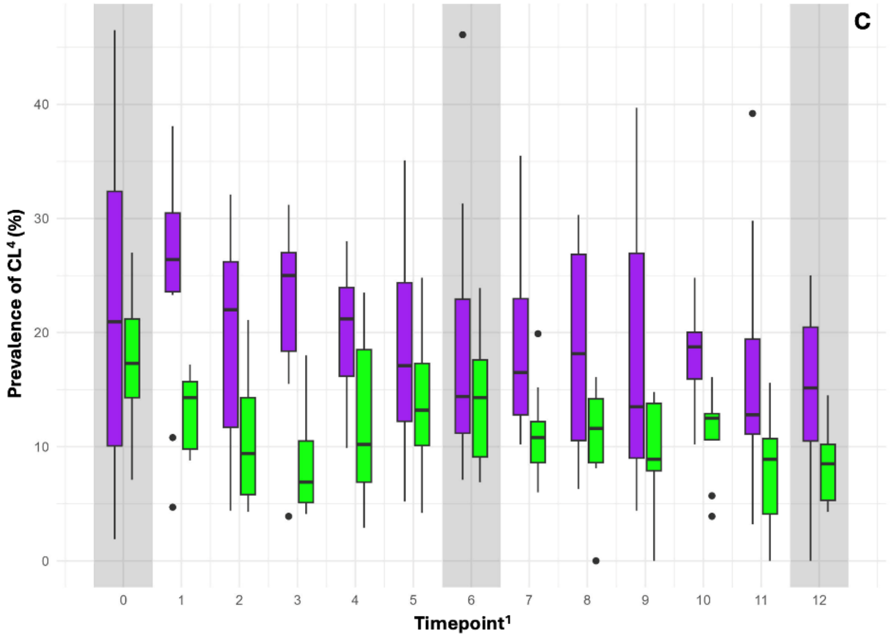
C
Prevalence of CL⁴ (%)
0
10
20
30
40
0 1 2 3 4 5 6 7 8 9 10 11 12
Timepoint¹
