# Supplementary material for: Successful implementation of a risk assessment and mitigation program to control bovine digital dermatitis at the herd-level
Source: Sci Rep. 2025 Aug 20;15:30577. doi: 10.1038/s41598-025-12093-5 (PMC12368109; doi:10.1038/s41598-025-12093-5)
Supplement: Supplementary file 3 — Supplementary Material 3 [file 41598_2025_12093_MOESM3_ESM.pdf]

Supplementary Table 2. Checklist used for assessment of farm-specific risk factors and consequent sanitation measures for bovine digital dermatitis (BDD) within the intervention group.

		**Comment**
**External biosecurity**		
M	Screening of newly introduced animals into the herd for BDD in the trimming chute	
M	Screening of re-introduced animals into the herd for BDD in the trimming chute	
M	Avoiding share of claw trimming equipment between farms; otherwise cleaning + disinfection before entering another farm	
**Internal biosecurity**		
M	Immediate treatment of all M-stages using Novaderma®, bandaged with 1-wk intervals until clinical cure	
M	Cleaning + disinfection of claw trimming tools after treating a BDD-affected animal	
M	Usage of single-use gloves after treating an animal with active BDD lesions	
M	Adaption of scraping frequency	
V	Avoiding of overcrowding	
V	Implementation of footbathing	
M	Topical disinfection of feet using a backpack sprayer	
M	Regular lameness scoring (min. 1x/wk)	
M	Regular screening for BDD (min. 1x every 14 d)	
M	Documentation + follow-up of treated/therapy-resistant animals	
V	Culling of therapy-resistant or recurrently affected animals	

M = mandatory; V = voluntary
